# A well-demarcated hyperpigmented plaque of the posterior neck

**DOI:** 10.1016/j.jdcr.2024.04.015

**Published:** 2024-04-23

**Authors:** Hannah L. Cornman, Emily K. Haque, Lo Tamburro, Marcia S. Driscoll

**Affiliations:** a Department of Dermatology, University of Maryland School of Medicine, Baltimore, Maryland; b Department of Pathology, University of Maryland School of Medicine, Baltimore, Maryland

**Keywords:** case report, dermatopathology, necrosis, pressure injury, surgery

## Case presentation

**Fig 1:**
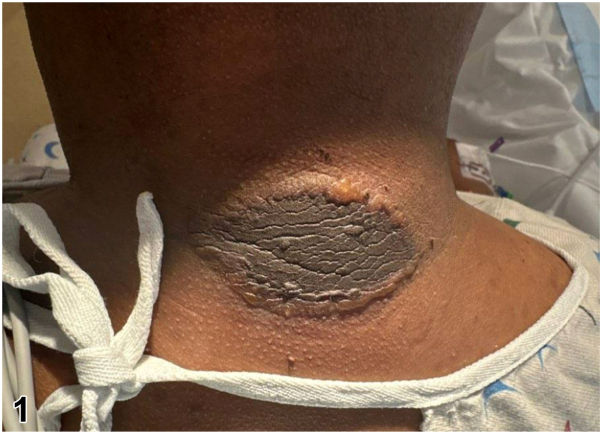

**Fig 2:**
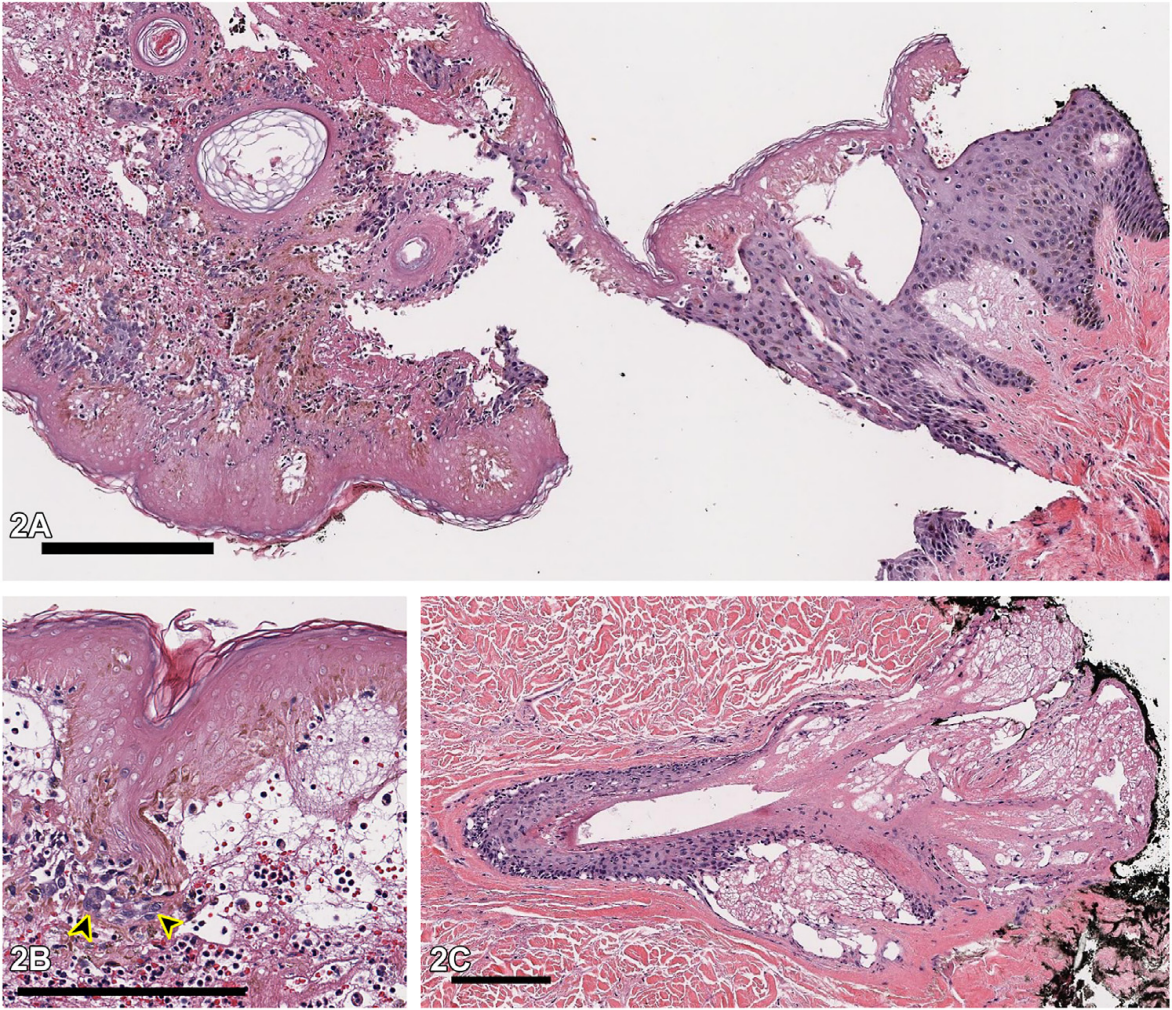

One day after a 12-hour surgery for medullary thyroid cancer, a 42-year-old diabetic man presented with an asymptomatic, well-demarcated, hyperpigmented neck plaque surrounded by honey-colored vesiculation (Fig 1). During surgery, the patient was in semi-Fowler position with a shoulder roll under his scapulae and a foam donut under his head. No history of similar lesions or new medications in the past year was reported. Inpatient medications were initiated within 24 to 48 hours of the lesion appearance, without anaphylactic symptoms. Histopathology of a 4.0-mm punch biopsy taken at the transition from normal to lesional skin revealed full-thickness epidermal necrosis, subepidermal clefting, sebaceous gland necrosis, intact stratum corneum, and minimal mixed dermal inflammation, without eosinophils (Fig 2, *A*-*C*). Direct immunofluorescence was not performed. Bacterial cultures of vesicular fluid were negative.

**Question 1: What is the most likely diagnosis?**

A. Allergic contact dermatitis (ACD)
B. Pressure-induced necrosis
C. Fixed drug eruption (FDE)
D. Lichen planus (LP)
E. Erythema multiforme (EM)

**Answers:**

A. ACD – Incorrect. Although the lesion’s timing of onset, geometric shape, and peripheral vesiculation might suggest ACD, ACD histology would show significant dermal inflammation and epidermal spongiosis. Additionally, ACD is usually painful and pruritic.
B. Pressure-induced necrosis – Correct. Pressure-induced necrosis, also called coma blisters, is caused by extended pressure to an area.^1^ Typical histopathologic findings include epidermal necrosis, intra- or subepidermal blistering, and necrosis of cutaneous adnexal structures, most classically eccrine coils, but also hair follicles and sebaceous glands.^2^ It typically manifests as bullae or vesicles but can also present as erythematous-to-violaceous patches or plaques.^3^ These lesions usually emerge within 24 to 72 hours of a pressure injury and self-resolve in 1 to 3 weeks.^3^
C. FDE – Incorrect. The lesion’s timing of onset and geometric shape could suggest FDE, and rapidly evolving FDE lesions can show epidermal and subepidermal blistering on histology; however, the lack of prominent dermal inflammation, including eosinophils, or vascular wall thickening makes FDE less likely.
D. LP – Incorrect. Like this plaque, LP lesions are often polygonal in shape, violaceous in color, and have an acute onset. The histological findings in this case though do not demonstrate lichenoid inflammation, making LP unlikely.
E. EM – Incorrect. EM may show epidermal necrosis and a subepidermal blister on histology, but would be expected to have significant dermal inflammation, with sparse or absent neutrophils. Moreover, EM characteristically presents as multiple targetoid lesions, not a single plaque.

**Question 2: What risk factor likely played the largest role in predisposing this patient to the development of this lesion?**

A. Ingestion of a particular medication
B. Infection with herpes simplex virus
C. Diabetes
D. Prolonged surgery
E. A history of atopic dermatitis

**Answers:**

A. Ingestion of a particular medication – Incorrect. Although an adverse drug reaction is a reasonable differential diagnosis, the drug history in this case makes it a less likely scenario. None of the patient’s home medications were new in the past year, the patient has no history of FDE-like lesions, and the lesion did not fit the picture for longer term complications like interstitial granulomatous drug reaction.
B. Infection with herpes simplex virus – Incorrect. Herpes simplex virus infection is associated with EM, but an EM diagnosis is less likely in this patient, for reasons discussed above.
C. Diabetes – Incorrect. The location, hyperpigmentation, and increased skin markings might be suggestive of acanthosis nigricans, and the patient is at risk for acanthosis nigricans due to his history of diabetes. However, acanthosis nigricans would have a chronic rather than acute onset.
D. Prolonged surgery – Correct. Postoperatively, prolonged surgery is the most important risk factor for pressure injury.^4^ It typically presents as ulcerations of the heel and sacral area among patients in semi-Fowler position, regions which are the focus of preventive efforts.^5^ However, in head and neck surgeries, pressure injuries affect the upper back in approximately 50% of patients.^4^ These risks were exemplified in our case by the 12-hour surgery and application of pressure to the lower neck by the foam donut or shoulder roll.
E. A history of atopic dermatitis – Incorrect. A history of atopic dermatitis may predispose patients to ACD or lichen simplex chronicus, but these diagnoses are less likely in this patient, for reasons discussed above.

**Question 3: What are the most appropriate next steps in management of this patient?**

A. Discontinue medications initiated in the hospital and observe for improvement
B. Treat the lesion with topical corticosteroids
C. Admit the patient to a burn unit and prevent dehydration with intravenous fluids
D. Reassurance and regular wound care
E. Conduct patch testing to verify the implicated allergen

**Answers:**

**Fig 3:**
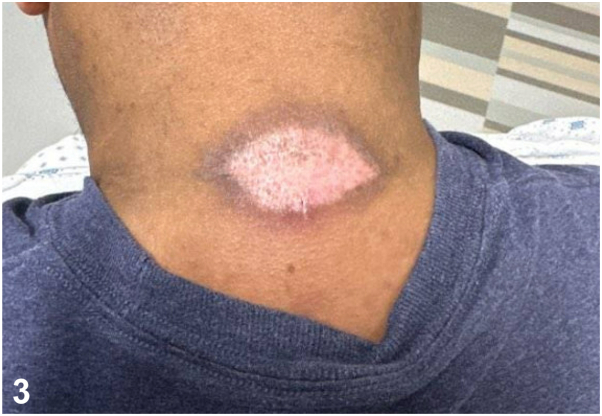
E. Conduct patch testing to verify the implicated allergen – Incorrect. This may be appropriate if the most likely diagnosis was ACD, but this diagnosis is less likely, for reasons described above.

A. Discontinue medications initiated in the hospital and observe for improvement – Incorrect. This would be appropriate management for an FDE or exanthematous drug reaction, but no medication discontinuation is required for pressure necrosis.
B. Treat the lesion with topical corticosteroids – Incorrect. LSC, EM, and ACD might benefit from topical corticosteroids, but pressure necrosis is not an inflammatory condition and therefore treatment with topical corticosteroids is not recommended.
C. Admit the patient to a burn unit and prevent dehydration with intravenous fluids – Incorrect. If the patient had findings concerning for Stevens Johnson Syndrome-toxic epidermal necrolysis, this would be appropriate. The singularity and stability of the skin lesion, its morphology, and the patient’s drug history are not concerning for Stevens Johnson Syndrome-toxic epidermal necrolysis.
D. Reassurance and regular wound care – Correct. Pressure-induced skin necrosis is a benign condition which is not related to any underlying infectious process or drug exposure.^1^^,^^3^ There is no known risk of progression to toxic epidermal necrolysis or other severe adverse cutaneous reaction.^1^^,^^3^ Most lesions will heal spontaneously within 1 to 3 weeks, as was the case in this patient (Fig 3). To promote healing and reduce scar development though, patients should be advised to avoid pressure to the wound site and engage in daily wound care such as removal of necrotic tissue, bandaging, and maintenance of a moist environment for wound healing.

## Conflicts of interest

None disclosed.
